# Disruption of mitochondrial unfolded protein response results in telomere shortening in mouse oocytes and somatic cells

**DOI:** 10.18632/aging.205543

**Published:** 2024-02-12

**Authors:** Mauro Cozzolino, Yagmur Ergun, Emma Ristori, Akanksha Garg, Gizem Imamoglu, Emre Seli

**Affiliations:** 1 Department of Obstetrics, Gynecology, and Reproductive Sciences, Yale School of Medicine, New Haven, CT 06510, USA; 2 IVIRMA Global Research Alliance, IVIRMA Roma, Rome, Italy; 3 IVIRMA Global Research Alliance, Fundacion IVI-IIS la Fe, Valencia, Spain; 4 IVIRMA Global Research Alliance, IVIRMA New Jersey, Basking Ridge, NJ 07920, USA; 5 IVIRMA Global Research Alliance, IVIRMA New Jersey, Marlton, NJ 08053, USA; 6 Department of Genetics, Yale School of Medicine, New Haven, CT 06510, USA; 7 Department of Metabolism, Digestion and Reproduction, Imperial College London, London, United Kingdom

**Keywords:** telomere length, aging, Clpp, mitochondrial dysfunction, unfolded protein response

## Abstract

Caseinolytic peptidase P (CLPP) plays a central role in mitochondrial unfolded protein response (mtUPR) by promoting the breakdown of misfolded proteins and setting in motion a cascade of reactions to re-establish protein homeostasis. Global germline deletion of *Clpp* in mice results in female infertility and accelerated follicular depletion. Telomeres are tandem repeats of 5’-TTAGGG-3’ sequences found at the ends of the chromosomes. Telomeres are essential for maintaining chromosome stability during somatic cell division and their shortening is associated with cellular senescence and aging. In this study, we asked whether the infertility and ovarian aging phenotype caused by global germline deletion of *Clpp* is associated with somatic aging, and tested telomere length in tissues of young and aging mice. We found that impaired mtUPR caused by the lack of CLPP is associated with accelerated telomere shortening in both oocytes and somatic cells of aging mice. In addition, expression of several genes that maintain telomere integrity was decreased, and double-strand DNA breaks were increased in telomeric regions. Our results highlight how impaired mtUPR can affect telomere integrity and demonstrate a link between loss of mitochondrial protein hemostasis, infertility, and somatic aging.

## INTRODUCTION

Human longevity has increased rapidly since the beginning of the 20^th^ century due to medical advances, societal changes, and improved quality of life, which subsequently has led to delayed childbearing [1, 2]. Consequently, the past two decades witnessed an increased interest in the investigation of the aging process. However, mechanisms regulating germ cell aging, and how they relate to somatic aging remain incompletely characterized.

Several mechanisms have been implicated in promoting cellular senescence, including telomere shortening [3] and mitochondrial dysfunction [4, 5]. Telomeres consist of tandem repeats of 5′-TTAGGG-3′ sequences that are located at the end of the chromosomes and play a crucial role in preserving chromosome stability. During each cell division, 20–50 telomeric base pairs cannot be replicated and are lost at the 5′-end of the newly synthesized DNA strand, leading to telomere shortening [6]. Decreasing telomere length acts as a “mitotic clock” for cellular senescence and aging. This is because telomere shortening that occurs with each round of cell division ultimately leads to chromosome ends becoming exposed and activates a DNA damage response, which results in a permanent mitotic arrest known as replicative senescence [7]. Telomere damage may also occur in non-dividing cells, such as oocytes, and may result from oxidative DNA damage to guanine-rich telomeric repeats when exposed to reactive oxygen species (ROS), as well as epigenetic, environmental, dietary, and lifestyle variables [8, 9].

Mitochondria on the other hand serve a crucial role in energy production, cellular metabolism, regulation of membrane potential, and apoptosis [10]. More recently, mitochondrial unfolded protein response (mtUPR), which ensures mitochondrial protein hemostasis by sensing mitochondrial (unfolded protein) stress, has been implicated in aging [11]. Activation of mtUPR contributes to enhanced longevity in experimental models [12–14], while a dysfunctional mtUPR may result in age-related accumulation of damaged proteins, decreased oxidative phosphorylation, and increased ROS (reviewed in [11]). Multiple mouse models of mitochondrial dysfunction also result in female infertility, further highlighting the complex relationship between mitochondrial function, aging and reproductive potential [5, 15, 16].

Caseinolytic peptidase P (CLPP) is a key regulator of mtUPR [17–20]. CLPP cleaves misfolded mitochondrial proteins that are then exported to the cytoplasm where they activate transcription factors that induce mtUPR (reviewed in [11]). Previous research has shown that global germline *Clpp* loss in female mice impairs oocyte maturation and two-cell embryo formation and causes blastocyst development failure, which eventually results in infertility [5, 21]. In addition, the absence of CLPP causes accelerated ovarian follicle depletion and results in a phenotype similar to diminished ovarian reserve and ovarian aging [5].

In this study, we aimed to investigate whether the infertility and ovarian follicular depletion phenotype observed in mice with global deletion of *Clpp* is associated with changes in somatic tissues that suggest accelerated aging. Our findings demonstrate that lack of *Clpp* causes shorter telomeres in oocytes and a number of somatic tissues of *Clpp*^−/−^ mice. These changes are associated with decreased expression of genes that regulate telomere length and stability.

## MATERIALS AND METHODS

### Animals

*Clpp*^+/−^ mice in a C57BL/6J background (Founder Line # IST13563G11) generated by Texas A&M Institute for Genomic Medicine (TIGM), an institute of AgriLife, and obtained from Georg Auburger, PhD (Goethe University Medical School, Frankfurt am Main, Germany) [21] were crossbred to obtain *Clpp*^−/−^ mice. Mice care, breeding, and experimental procedures were conducted according to the Yale University Animal Research requirements, by using protocols approved by the Institutional Animal Care and Use Committee (Protocol #2020-11207). Genotyping was carried out using the methods previously described [22].

### Collection of oocytes and cumulus cells

To obtain immature (germinal vesicle-stage, GV) oocytes, 2-, 6-, and 9-month-old *Clpp*^−/−^ and wild type (WT) female mice were injected intraperitoneally with 10IU pregnant mare’s serum gonadotropin (PMSG, Sigma, St. Louis, MO, USA). After 44 hours of PMSG injection, ovaries were removed and punctured with a 26-gauge needle in M2 medium (Sigma, St. Louis, MO, USA) supplemented by 10 μM milrinone (Sigma, St. Louis, MO, USA) to prevent meiotic resumption.

To collect mature oocytes arrested in the metaphase of the second meiotic division (MII), 10IU of human chorionic gonadotropin (hCG; Sigma, St. Louis, MO, USA) was injected 48 h hours after the PMSG (Sigma, St. Louis, MO, USA). After 14–16 hours of hCG injection, unfertilized MII oocytes were collected from oviducts. Oocytes were stripped from cumulus cells with a mouth pipette and collected in individual tubes.

### Blood and tissue collection

Blood samples (0.5 ml) were collected from each mouse (*n* = 5) through intracardiac puncture with a 18-gauge needle. After blood collection, mice were perfused with normal saline through a needle in the left ventricle. After perfusion, tissues (heart, liver, spleen, lung, kidney, uterus, and ovaries) were dissected and washed in Dulbecco’s Phosphate Buffer Saline (DPBS) for 10 seconds. Tissues were stored at −80°C until further experiments.

### Telomere length measurement

GV and MII stage oocytes (collected from 10 mice for each genotype at each timepoint, and pooled as 2 mice per sample) were collected from 2-, 6-, and 9-month-old *Clpp*^−/−^ mice and compared to WT. DNA extraction from oocytes and cumulus cells was performed using the QIAmp DNA Micro Kit (Qiagen, Valencia, CA, USA), and DNA extraction from white blood cells (WBC) was conducted with DNA Isolation Kit for Mammalian Blood (Roche, Basel, Switzerland) according to manufacturer’s protocol and both concentrations were quantified using Qubit 3.0 (Life Technologies).

Average telomere length was measured from total genomic mouse DNA using a real-time quantitative PCR method previously described [16]. The average telomere length ratio was obtained by quantifying telomeric DNA with specially designed primer sequences and dividing that amount by the quantity of a single-copy gene, acidic ribosomal phosphoprotein PO (36B4) gene. Forward and reverse telomere and 36B4 primers are shown in Supplementary Table 1. Each reaction included 10 μl iQ™ SYBR^®^ Green Supermix, (Bio-Rad), 400 nM of each primer, 1 ng genomic DNA, and enough double-distilled H_2_O to complete the volume in 20-μl reaction. PCR reactions were performed on the iCycler iQ real-time PCR detection system (Bio-Rad, Hercules, CA, USA). For each PCR reaction, a standard curve was generated by serial dilutions of known amounts of DNA from the same tissues. The telomere signal was normalized to the signal from the single-copy gene to generate a T/S ratio indicative of relative telomere length. The relative input amount of the telomere PCR then was divided by the relative input amount of the 36B4 PCR of the same sample. Each real-time PCR experiment was repeated a minimum of three times.

### Quantitative reverse-transcription polymerase chain reaction (qRT-PCR)

Total RNA was obtained from 20 oocytes per mouse using RNAqueous Microkit (Ambion, Austin, TX, USA) and was treated for genomic DNA contamination using DNase I (Ambion). Reverse transcription was performed using the RETROscript kit (Ambion) in two steps: first, template RNA and oligo(dT) primers were incubated at 85°C for 3 min to eliminate any secondary structures, and then the buffer and enzyme were added and the reaction was carried out at 42°C for 1 h. qPCR was carried out on an iCycler (Bio-Rad Laboratories). Each 10-μl PCR reaction contained 5 μl of iQ™ SYBR^®^ Green Supermix (Bio-Rad Laboratories), 3 μl of H2O, 0.5 μl of each primer, and 1 μl of cDNA. The 2^−ΔΔCT^ (cycle threshold) method was used to calculate relative expression levels after normalization to *β-actin* or *Gapdh* levels. Samples were assayed in triplicate and each experiment was repeated at least three times using individual animals from each genotype. The primers used for real-time PCR reactions are reported in Supplementary Table 1.

### Immunofluorescent staining

For immunofluorescent staining, cumulus oophorus complexes (COCs) containing GV stage oocytes were collected and fixed with 4% paraformaldehyde (Sigma, St. Louis, MO, USA) in Dulbecco’s Phosphate Buffer Saline (DPBS) for 5 min, then washed three times in 1X DPBS. They were placed in 0.5% Triton X-100 in DPBS at room temperature for 10 minutes, then washed in DPBS for 5 minutes. After blocking in 3% BSA (Sigma, St. Louis, MO, USA) at room temperature for 1 h, COCs were incubated overnight at 4°C with rat anti-TRF1 monoclonal antibody (Abcam, Cambridge, UK Cat# ab192629, 1:100) or mouse anti-H2AX monoclonal antibody (Sigma, St. Louis, MO Cat# 05-636-25UG 1:100) as a primary antibody. After three washes with 1X DPBS for 10 minutes, COCs were incubated with Alexa Fluor 488-conjugated goat anti-mouse antibody (1:400) or Alexa Fluor 568-conjugated goat anti-rat antibody (1:400) for 1 h at room temperature. Finally, they were mounted with 4′,6-diamidino-2-phenylindole (DAPI;1:1000) (Life Technologies, Carlsbad, CA, USA) on glass slides and stored at 4°C until imaging. Images were captured on Leica SP8 spectral scanning confocal microscope.

H2AX and TRF1 fluorescent intensities were quantified using ImageJ, and measured as corrected total cell fluorescence (CTCF), in arbitrary units, as described by Bora et al. [23], using the following formula: CTCF = Integrated density – Area of selected nucleus x Mean fluorescence of the background readings. Co-localization results were obtained with Volocity software (PerkinElmer) and analyzed using Pearson’s correlation coefficient. Results were statistically analyzed using the Mann–Whitney test.

### Statistical analysis

Quantitative data are expressed as mean ± standard deviation (SD). The student’s *t*-test was used to analyze the statistical significance between the two groups and ANOVA was used for multiple groups. Fluorescent intensity analyses were performed as described above. Data are representative of at least three independent experiments unless otherwise specified. All statistical analyses were done using GraphPad Prism software version 9 and significance was assessed at *p* < 0.05.

## RESULTS

### Telomere length in *Clpp*^−/−^ mice oocytes, ovaries and somatic cells and tissues

**Figure 1:**
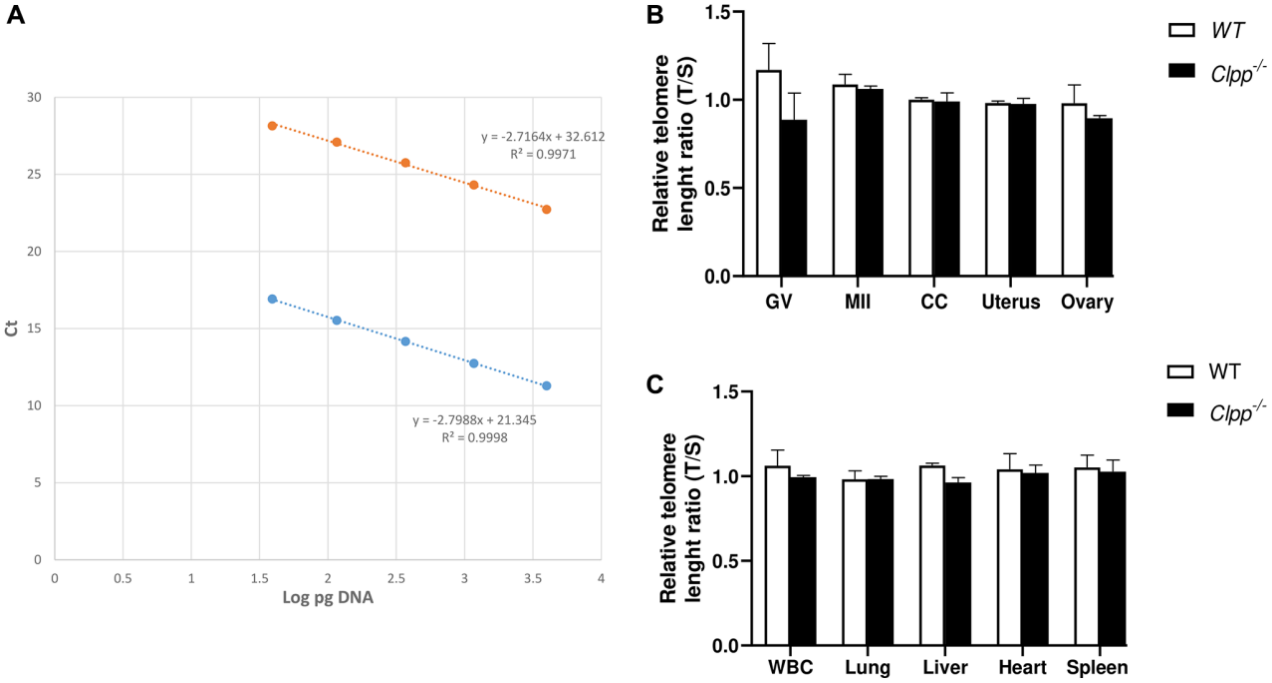
**Telomere length in oocytes, ovaries, and somatic tissues of 2-month-old *Clpp*^−/−^ and WT mice.** (**A**) The standard curve was generated by serial dilution of known amounts of DNA to calculate relative DNA concentrations (log DNA) from Ct values of the qPCR products. Blue dots: telomere; orange dots: 36B4 single copy gene (control). The correlation regression equation and coefficients (R2) of Ct versus log DNA are shown. (**B**, **C**) The relative telomere lengths of GV and MII oocytes, ovaries and somatic cells and tissues are represented as ratios of T/S. Abbreviations: GV: Germinal vesicle; CC: Cumulus cells; WBC: White blood cells. Data presented as mean ± SD. ^**^p < 0.01, ^*^p < 0.05 using *t*-test. The telomere length assessment was repeated twice, using five mice (ten GVs from each) in each group per experiment.

In young (2-month-old) *Clpp*^−/−^ mice, telomere length in GV and MII oocytes was similar to WT (1.16 ± 0.17 vs. 0.88 ± 0.38, *p* = 0.08 and 1.08 ± 0.08 vs. 1.06 ± 0.24, *p* = 0.52, respectively) (Figure 1A, 1B). Telomere length of cumulus cells (CCs) and other somatic cells in 2-month-old *Clpp*^−/−^ mice were also found to be similar compared to WT (Figure 1B, 1C).

**Figure 2:**
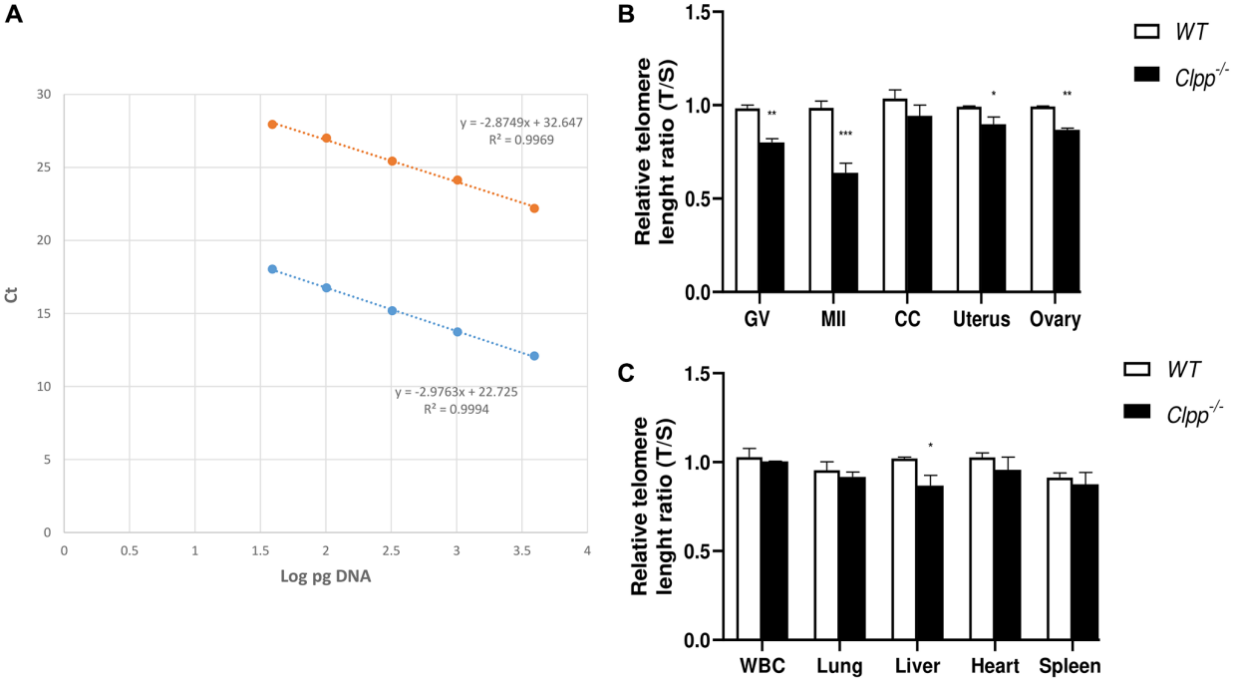
**Telomere length in oocytes, ovaries, and somatic tissues of 6-month-old *Clpp*^−/−^ and WT mice.** (**A**) The standard curve was generated by serial dilution of known amounts of DNA to calculate relative DNA concentrations (log DNA) from Ct values of the qPCR products. Blue dots: telomere; orange dots: 36B4 single copy gene (control). The correlation regression equation and coefficients (R2) of Ct versus log DNA are shown. (**B**, **C**) The relative telomere lengths of GV and MII oocytes, ovaries and somatic cells and tissues are represented as ratios of T/S. Abbreviations: GV: Germinal vesicle, CC: Cumulus cells, WBC: White blood cells. Data presented as mean ± SD. ^**^p < 0.01, ^*^p < 0.05 using *t*-test. The telomere length assessment was repeated twice, using five mice (ten GVs from each) in each group per experiment.

To assess the impact of aging, samples from 6-, and 9-month-old *Clpp*^−/−^ and WT mice were analyzed. Telomere length of GV and MII oocytes from 6-month-old *Clpp*^−/−^ mice were significantly shorter compared to WT (0.98 ± 0.01 vs. 0.80 ± 0.03, *p* < 0.0001 and 0.99 ± 0.02 vs. 0.69 ± 0.04, *p* = 0.001, respectively) (Figure 2A, 2B). Telomere length was also shorter in the uterus, ovary, and liver of 6-month-old *Clpp*^−/−^ mice compared to WT (0.99 ± 0.46 vs. 0.89 ± 0.41, *p* = 0.04; 0.99 ± 0.02 vs. 0.87 ± 0.01, *p* = 0.002 and 1.02 ± 0.005 vs. 0.86 ± 0.04, *p* = 0.04, respectively) (Figure 2C). In CCs, white blood cells (WBCs), lung, heart and spleen, mean telomere length was shorter in *Clpp*^−/−^, however, the difference was not statistically significant compared to WT mice (Figure 2B, 2C).

**Figure 3:**
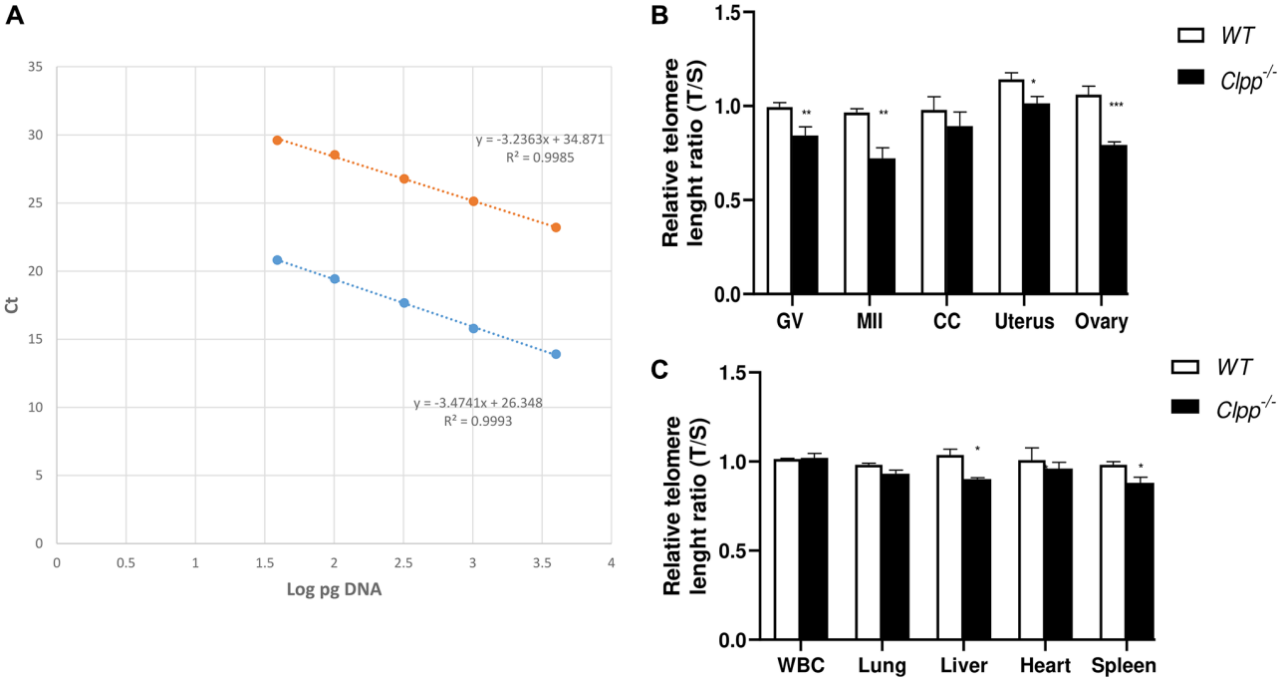
**Telomere length in oocytes, ovaries, and somatic tissues of 9-month-old *Clpp*^−/−^ and WT mice.** (**A**) The standard curve was generated by serial dilution of known amounts of DNA to calculate relative DNA concentrations (log DNA) from Ct values of the qPCR products. Blue dots: telomere; orange dots: 36B4 single copy gene (control). The correlation regression equation and coefficients (R2) of Ct versus log DNA are shown. (**B**, **C**) The relative telomere lengths of GV and MII oocytes, ovaries and somatic cells and tissues are represented as ratios of T/S. Abbreviations: GV: Germinal vesicle; CC: Cumulus cells; WBC: White blood cells. Data presented as mean ± SD. ^**^p < 0.01, ^*^p < 0.05 using *t*-test. The telomere length assessment was repeated twice, using five mice (ten GVs from each) in each group per experiment.

In 9-month-old mice, telomere length in GV and MII oocytes was also significantly shorter in *Clpp*^−/−^ mice compared to WT (1.0 ± 0.07 vs. 0.84 ± 0.05, *p* = 0.01 and 0.99 ± 0.04 vs. 0.89 ± 0.02, p = 0.01, respectively) (Figure 3A, 3B). In somatic tissues, telomere length was significantly shorter in the uterus (1.14 ± 0.03 vs. 1.01 ± 0.03, *p* = 0.01), ovary (1.06 ± 0.04 vs. 0.79 ± 0.01, *p* = 0.004), liver (1.03 ± 0.02 vs. 0.90 ± 0.06, *p* = 0.01) and spleen (0.98 ± 0.01 vs. 0.88 ± 0.02, *p* = 0.01). Conversely, while the mean telomere length was also lower in CCs, WBCs, lung, and heart in *Clpp*^−/−^ mice compared to WT, the difference was not statistically significant (Figure 3B, 3C). The telomere length assessment was repeated twice, using five mice (ten GV for each) in each group per experiment.

### Expression of telomere-associated genes in *Clpp*^−/−^ oocytes

TRF1, TRF2 and POT1a are shelterin protein complex molecules involved in maintaining telomere DNA integrity and stability. Their deficiency causes DNA damage response and the accumulation of DNA repair factors [24, 25]. H2AX is a minor nucleosomal histone protein [26, 27]. H2AX becomes phosphorylated at sites of double-stranded DNA breaks and antibodies against the serine-139 phosphorylated form of H2AX allow microscopic detection of individual double-stranded breaks in the DNA [28].

**Figure 4:**
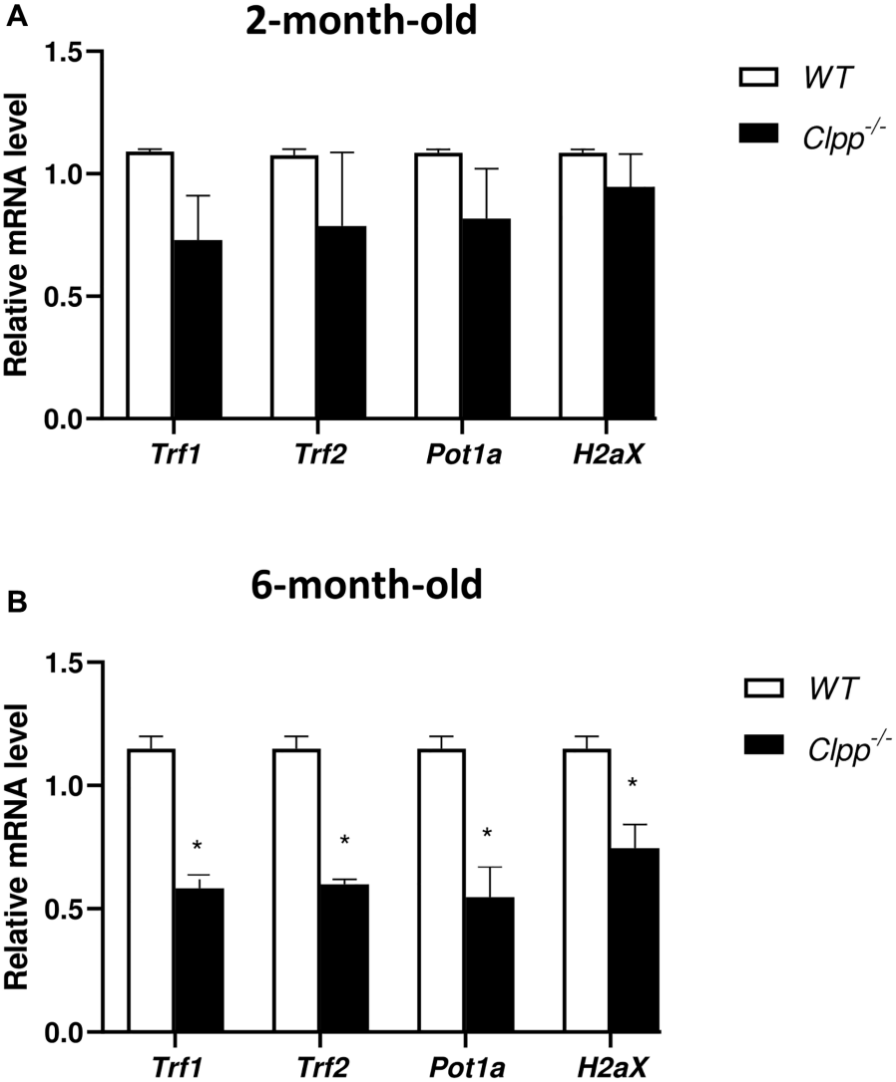
**Expression of telomere-associated genes in *Clpp*^−/−^ and WT oocytes.** Expression of telomere-associated genes was assessed using qRT-PCR in GV oocytes collected from 2-month-old (**A**) and 6-month-old (**B**) *Clpp*^−/−^ and WT mice. Data presented as mean ± SD with *t*-test (^**^p < 0.01, ^*^p < 0.05). Experiments repeated twice, using five mice (ten GVs from each) in each group per experiment.

We first used qRT-PCR to quantify *Trf1, Trf2, Pot1a, and H2Aax* expression in our model. In 2-month-old mice, there was no significant difference between *Clpp*^−/−^ and WT mice for *Trf1, Trf2, Pot1a, or H2ax* expression (Figure 4A). Conversely, in the 6-month-old mice there was a significant decrease in *Clpp*^−/−^ compared to WT mice for *Trf1* (0.58 ± 0.06 vs. 1.12 ± 0.04, *p* = 0.03), *Trf2* (0.59 ± 0.07 vs. 1.11 ± 0.02, *p* = 0.008), *Pot1a* (0.61 ± 0.32 vs. 1.13 ± 0.03, *p* = 0.05), and *H2Ax* (0.67 ± 0.12 vs. 1.11 ± 0.02, *p* = 0.03) (Figure 4B). The qRT-PCR experiments were repeated twice, using five mice (ten GV for each) in each group per experiment.

**Figure 5:**
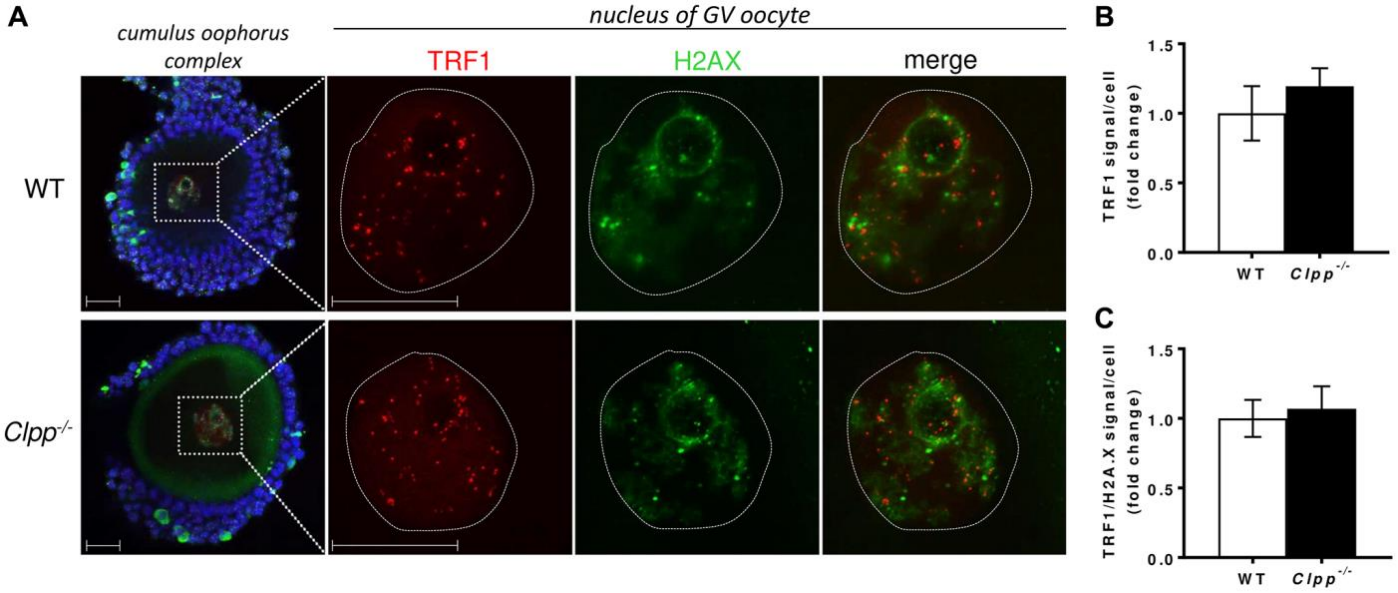
**Representative confocal images of TRF1 expression and TRF/H2AX co-localization in cumulus oophorus isolated from 2-month-old wild-type and *Clpp*^−/−^ mice.** (**A**) Immunofluorescence double staining of TRF1 (red) and H2AX (green) in cumulus oophorus complexes of 2-month-old WT and *Clpp*^−/−^ mice. Nuclear area of GV oocytes is highlighted by a dotted line. Nuclei were stained for TRF1 (red) and H2AX (green). DAPI (Blue) was used to stain nuclei (blue). The highlighted box in *Clpp*^−/−^ sample shows co-localization of TRF1 and H2AX (white arrow). Scale bar = 25 μm. DAPI was used to stain nuclei (blue). (**B**) Quantitative analysis of TRF1 immunofluorescence in WT and *Clpp*^−/−^ GV oocytes. (**C**) Quantitative analysis of co-localization of TRF1 and H2AX in WT and *Clpp*^−/−^ GV oocytes. Data presented as mean ± SD with *t*-test (^**^p < 0.01, ^*^p < 0.05). Experiments repeated twice, using three mice (five GVs from each) in each group per experiment.

**Figure 6:**
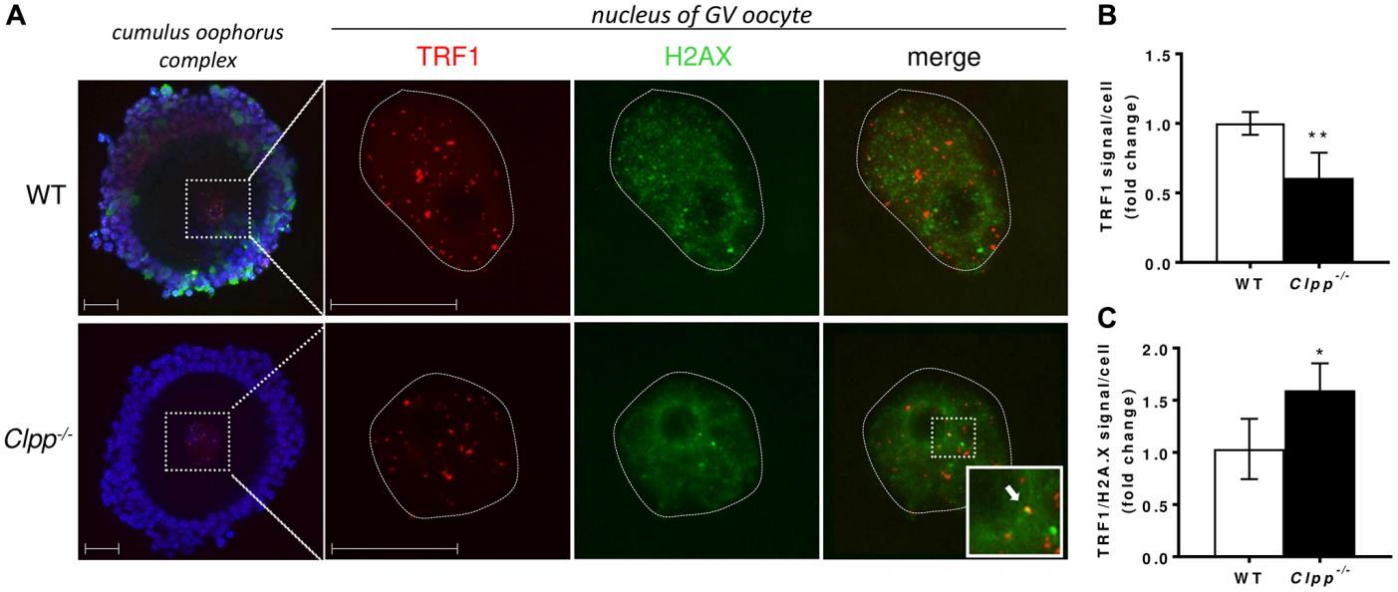
**Representative confocal images of TRF1 expression and TRF/H2AX co-localization in cumulus oophorus isolated from 6-month-old wild-type and *Clpp*^−/−^ mice.** (**A**) Immunofluorescence double staining of TRF1 (red) and H2AX (green) in cumulus oophorus complexes of 6-month-old *Clpp*^−/−^ and WT mice. Nuclear area of GV oocytes is highlighted by a dotted line. Nuclei were stained for TRF1 (red) and H2AX (green). DAPI (Blue) was used to stain nuclei (blue). The highlighted box in *Clpp*^−/−^ sample shows co-localization of TRF1 and H2AX (white arrow). Scale bar = 25 μm. (**B**) Quantitative analysis of TRF1 immunofluorescence in WT and *Clpp*^−/−^ GV oocytes. (**C**) Quantitative analysis of co-localization of TRF1 and H2AX in WT and *Clpp*^−/−^ GV oocytes. Data presented as mean ± SD with *t*-test (^**^p < 0.01, ^*^p < 0.05). Experiments repeated twice, using three mice (five GVs from each) in each group per experiment.

To further investigate the accelerated telomere damage in GV oocytes, we performed co-immunofluorescence staining of TRF1 and H2AX in 2- and 6-month-old *Clpp*^−/−^ and WT mice. In the nuclei of 2-month-old *Clpp*^−/−^ GV oocytes, there was no significant difference in TRF1 (1.0 ± 0.13 vs. 1.19 ± 0.16, *p* = 0.23) (Figure 5A, 5B) or H2AX (1.0 ± 0.23 vs. 1.03 ± 0.35, *p* = 0.18) (Supplementary Figure 1) immunofluorescence intensity compared to WT. In addition, TRF1 did not co-localize with H2AX in *Clpp*^−/−^ oocytes, indicating the absence of telomere damage (1.0 ± 0.28 vs. 1.33 ± 0.25, *p* = 0.16) (Figure 5C). In 6-month-old mice, we found the expression of TRF1 to be significantly decreased in oocytes’ nuclei compared to WT (1.0 ± 0.08 vs. 0.60 ± 0.18, *p* = 0.005) (Figure 6A, 6B). H2AX immunofluorescence intensity was also lower, although the difference did not reach statistical significance (1.0 ± 0.41 vs. 0.64 ± 0.38, *p* = 0.09) (Supplementary Figure 1). Additionally, there was an increased co-localization of TRF1 with H2AX (0.98 ± 0.17 vs. 1.33 ± 0.11, *p* = 0.006) indicating telomeric damage in the *Clpp*^−/−^ oocytes (Figure 6C). Representative images of GV oocytes isolated from 6-month-old WT mice that were negative controls for TRF1 and H2AX immunofluorescence are shown in Supplementary Figure 2. The immunofluorescence experiments were repeated twice, using three mice (five GV for each) in each group per experiment.

## DISCUSSION

CLPP is essential for mtUPR as it promotes the breakdown of misfolded proteins within the mitochondria and initiates a series of reactions to eliminate the detrimental impact of mitochondrial stressors and to re-establish protein homeostasis [11]. Studies using knockout mouse models demonstrated that CLPP is required for female fertility [5, 21]. In addition, global germline deletion of *Clpp* results in the activation of the mTOR (mammalian target of rapamycin) pathway and causes accelerated loss of ovarian follicular reserve, highlighting the role of CLPP in female reproductive competence and senescence [5]. In this study, we asked whether the infertility and ovarian aging phenotype caused by global germline deletion of *Clpp* is associated with somatic aging, and tested telomere length in young and aging mice gametes, gonads and somatic tissues. We found shortening of telomeres in both oocytes and somatic tissues at 6 and 9 months. In addition, expression of several genes associated with telomere integrity were decreased, and double strand DNA breaks were increased in telomeric regions. Our findings demonstrate how loss of mitochondrial protein homeostasis may accelerate telomere shortening in oocytes and somatic cells, and provide a link between reproductive and somatic aging.

A number of plausible mechanisms could be linking mitochondrial dysfunction and telomere shortening. Oxidative stress causes cell death and/or senescence, and as a result, rapid cell divisions occur in the surrounding essential cells as a protective strategy, which leads to telomere shortening [29, 30]. Reciprocally, in tissues with short telomeres, mitochondrial quantity and oxidative phosphorylation capacity are impaired, resulting in decreased ATP synthesis, decreased metabolic capacity, impaired gluconeogenesis, and elevated ROS levels [31, 32]. We have previously reported that global knockout of *Clpp* resulted in increased ROS levels in GV oocytes [5], strengthening the association between increased ROS and telomere shortening in this model.

Telomere shortening occurs upon cell division and a measurable shortening of telomeres requires repetitive cycles of cell division. It would be extremely challenging to maintain cellular characteristics of origin (e.g. liver or spleen) in a primary cell culture through the high number of cell divisions needed to achieve a measurable change in telomere length. Therefore, like others before us [33], we used an *in vivo* animal model, allowing us to study cells in their respective tissues after rounds of mitosis, and compare Clpp^−/−^ to WT. In 6- and 9-month-old *Clpp*-deficient mice, we found shorter telomere length in the liver when compared with WT, suggesting that the impaired mitochondrial function within the liver in *Clpp*^−/−^ mice could be triggering hepatic cellular changes that culminate in telomere shortening. Highly proliferative tissues, including the hematopoietic and immune systems, exhibit impaired proliferative capacity in later generations of the telomerase RNA component (TERC)-deficient mice [34, 35]. Telomerase complex gene mutations have been linked to rare human diseases such as Dyskeratosis Congenita (DKC) and Idiopathic Pulmonary Fibrosis [36], both of which are characterized by accelerated telomere shortening and organ failure. Patients with such diseases had an increased frequency of liver pathologies such as fibrosis and cirrhosis [37, 38]. Similarly, two studies investigated the frequency of telomerase mutations in patients with sporadic cirrhosis compared to healthy controls and demonstrated mutation missense mutations in the Telomerase Reverse Transcriptase (TERT) and TERC genes in diseased patients [39].

It is noteworthy that we found telomere length to be shorter in the liver and spleen of 9-month-old *Clpp*-deficient mice compared to WT, while the difference did not reach statistical significance in samples of lung and heart. *Clpp* is predominantly expressed in tissues that have an active metabolism and contain a higher number of mitochondria such as the skeletal muscle and liver [40], whereas it is detected at lower levels in lung and kidney. It is plausible that mitochondrial dysfunction in tissues where CLPP is most abundant is more likely to result in shortened telomeres.

Global deletion of *Clpp* resulted in mitochondrial dysfunction in oocytes by decreasing ATP production, expression levels of Electron Transport Chain (ETC) enzymes and increased levels of ROS [5]. Besides, the lack of *Clpp* affected the reproductive phenotype. Mice demonstrated a decrease in the number of mature oocytes and 2-cell embryos, and no blastocysts, resulting in infertility. In addition, they had accelerated follicular depletion and a phenotype consistent with diminished ovarian reserve [5]. In the current study, oocyte telomeres are shortened in at 6- and 9-month-old mice models with global deletion of *Clpp.* Consistent with our findings, when guanine-rich telomeric repeats are exposed to oxidative stress including ROS, they undergo oxidative DNA damage that shortens telomeres in non-dividing cells like oocytes, thereby potentially accelerating aging [41].

Global deletion of *Clpp* is also associated with spindle abnormalities and decreased ability to complete *in vitro* maturation of GV stage oocytes [5]. In the current study, shorter telomeres in 6- and 9-month-old *Clpp*^−/−^ mice oocytes suggest that the reproductive phenotype of *Clpp*^−/−^ mice may be, in part, due to oocyte telomere shortening. Indeed, telomeres play an important role in the regulation of chromosomal motions during meiosis, including bouquet creation at the leptotene stage, homologous pairing, and interaction with microtubules and spindles [42, 43]. Short telomeres are linked to infertility, aberrant spindles, and misaligned metaphase chromosomes in oocytes of telomerase-deficient mice [44]. Similarly, telomere length in human unfertilized oocytes was associated with the morphological quality of the embryos generated from sibling oocytes from the same cohort, and subsequent pregnancy outcomes [45, 46].

The impact of telomere length on oocyte function has been explored in animal and human studies [45–47]. Meanwhile, the role telomere length in cumulus/granulosa cell function remains to be further characterized, and existing data does not suggest this as a key regulatory mechanism. In the context of mtUPR, while global *Clpp* knockout was associated with infertility [5], granulosa cell-specific targeted deletion of *Clpp* did not affect reproduction [48]. In keeping with these findings, we did not find cumulus/granulosa cell telomere length to be shortened in *Clpp*^−/−^ mice. This is not surprising as recent studies investigating the telomere length and epigenetic aging markers in human cumulus/granulosa cells did not see a difference when comparing younger versus older reproductive age women or those with good versus poor ovarian response [49–51].

A number of telomere proteins, including TRF1, TRF2, and POT1a, regulate telomere stability and length [52]. Zhang et al. revealed that mice with targeted deletion of mitochondrial fusion protein Mitofusin 2 (MFN2) have defective oocyte maturation, subfertility, shortened telomeres, and decreased TRF1 expression in oocytes [16]. They also showed co-localization of TRF1 with DNA repair factor 53BP1, suggesting DNA damage. Our findings are similar in that *Trf1*, *Trf2*, and *Pot1a* expression is decreased in 6-month-old *Clpp*^−/−^ mice oocytes, and TRF1 co-localizes with DBA repair factor H2AX. Collectively these two studies show a consistent effect of mitochondrial dysfunction impacting dysregulated telomere mechanisms, thereby leading to DNA damage and telomere shortening.

In this study, we expanded our understanding of the role of *Clpp* and mtUPR in female reproduction and aging by characterizing telomere shortening and damage in a mouse model with global deletion of *Clpp.* As reported previously, the lack of *Clpp* results in functional mitochondrial abnormalities, infertility, and ovarian follicular depletion/aging. The current study demonstrates how this mitochondrial pathway, when impaired, may also promote somatic aging. In addition, our findings provide a preliminary understanding of how mitochondrial and telomeric aging mechanisms may interact to accelerate reproductive and somatic aging. Further studies are needed to delineate the intricate interactions between these two aging pathways and to determine whether these could be exploited to delay or reverse ovarian (or somatic) aging.

## Supplementary Materials

Supplementary Figures

Supplementary Table 1
