# Supplementary material for: Disruption of mitochondrial unfolded protein response results in telomere shortening in mouse oocytes and somatic cells
Source: Aging (Albany NY). 2024 Feb 12;16(3):2047–60. doi: 10.18632/aging.205543 (PMC10911389; doi:10.18632/aging.205543)
Supplement: Supplementary Figures [file aging-16-205543-s001.pdf]

SUPPLEMENTARY FIGURES

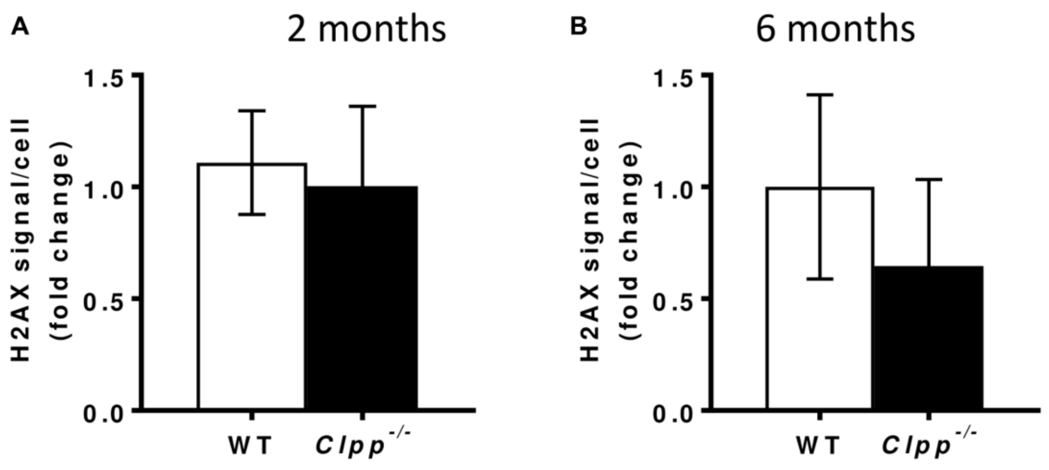

Supplementary Figure 1. Quantification of H2AX fluorescence intensity in 2-month (**A**) and 6-month (**B**) old mice.

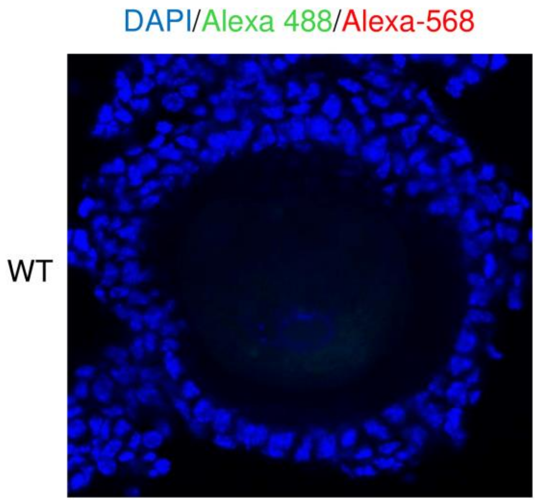

Supplementary Figure 2. Representative confocal image of GV oocyte isolated from a 6-month-old WT mice. Negative control for TRF1 and H2AX immunofluorescence. Samples were incubated only with secondary antibodies to check for aspecific staining.
