# Supplementary material for: Disruption of mitochondrial unfolded protein response results in telomere shortening in mouse oocytes and somatic cells
Source: Aging (Albany NY). 2024 Feb 12;16(3):2047–60. doi: 10.18632/aging.205543 (PMC10911389; doi:10.18632/aging.205543)
Supplement: Supplementary Table 1 [file aging-16-205543-s002.pdf]

SUPPLEMENTARY TABLE

Supplementary Table 1. The list of primers used for quantitative RT-PCR.

Genes	Primers sequences
Tel	F: CGGTTTGTTTGGGTTTGGGTTTGGGTTTGGGTTTGGGTTTGGGTT<br>R: GGCTTGCCTTACCTTTACCCTTACCCTTACCCTTACCCT
36B4	F: ACTGGTCTAGGACCCGAGAAG<br>R: TCAATGGTGCCTCTGGAGATT
Trf1	F: TCTAAGGATAGGCCAGATGCCA<br>R: CTGAAATCTGATGGAGCACGTC
Trf2	F: TCAGCTGCTTCAAGTACAATGAG<br>R: GGTTCTGAGGCTGTCTGCTT
Pot1a	F: TCTTCGGTTGTGGAAAGCCT<br>R: TGTTTGATGAAAAATCCTCTCACAG
H2Ax	F: TCGGGCCGCGGCAAGACTGGCGGCAA<br>R: GTACTCCTGGGAGGCCTGGGTGGCCTT

Abbreviations: Tel: Telomere length; 36B4: acidic ribosomal phosphoprotein P0; Trf1: telomeric repeat binding factor 1; Trf2: telomeric repeat binding factor 2; Pot1a: Protection of telomeres protein 1a; H2Ax: H2A histone family member X.
